# Ulipristal acetate simultaneously provokes antiproliferative and proinflammatory responses in endometrial cancer cells

**DOI:** 10.1016/j.heliyon.2021.e08696

**Published:** 2021-12-29

**Authors:** Ranka Kanda, Yuko Miyagawa, Osamu Wada-Hiraike, Haruko Hiraike, Kazunori Nagasaka, Eiji Ryo, Tomoyuki Fujii, Yutaka Osuga, Takuya Ayabe

**Affiliations:** a Department of Obstetrics and Gynecology, Teikyo University School of Medicine, Tokyo, Japan; b Department of Obstetrics and Gynecology, The University of Tokyo, 7-3-1 Hongo Bunkyo-ku, Tokyo 113-8655, Japan

**Keywords:** Ulipristal acetate, Selective progesterone receptor modulator, Progesterone receptor modulator–associated endometrial change, Apoptosis, Proinflammatory cytokine

## Abstract

Ulipristal acetate (UPA), a selective progesterone receptor modulator, is used for the treatment of uterine fibroids and selectively inhibits the proliferation and inflammation of leiomyoma cells. As few studies have focused on the molecular biological mechanism of UPA in Ishikawa endometrial cancer cells, we aimed to identify the effects of UPA on these cells. Ishikawa cells were treated with different concentrations of UPA. Cell viability and colony formation assays were performed to assess the growth of cancer cells, whereas invasion and migration assays were used to measure cell motility and invasiveness. Western blotting, caspase 3/7 assay, TUNEL assay, and flow cytometry were performed to analyze apoptosis. Moreover, expression levels of the proinflammatory cytokines oncostatin M, its receptor, interleukin 6, and interleukin 8 were examined using quantitative real-time PCR. UPA decreased cell viability and growth, thereby inhibiting cell migration and invasion via induction of apoptosis. Contrary to expectation, stand-alone application of UPA increased the expression of the proinflammatory cytokines but concomitant use of UPA and the estrogen receptor antagonist ICI 182,720 decreased it. These data revealed a novel dual role of UPA: It could attenuate cell growth via activation of apoptosis while simultaneously provoking the activation of proinflammatory cytokines in endometrial cancer cells. These indicate that the combination of UPA and an estrogen receptor antagonist may be useful in suppressing the secretion of proinflammatory cytokines by UPA alone.

## Highlights

• A selective progesterone receptor modulator ulipristal acetate, used for the treatment of uterine fibroids and selectively inhibits the proliferation and inflammation of leiomyoma cells decreased cell viability and growth in Ishikawa endometrial cancer cells.
• Ulipristal acetate induced apoptosis in endometrial cancer cells and activated oncostatin M, IL-6, IL-8 known as proinflammatory cytokine.
• Combination of Ulipristal acetate and estrogen receptor antagonist downregulated proinflammatory cytokine in endometrial cancer cells.

## 1 Introduction

Progesterone controls several reproductive functions, such as endometrial decidual alteration, regulation of implantation, mammary epithelial development, and regulation of GnRH pulse secretion, and is one of the female hormones secreted by the corpus luteum, which is mainly composed of granulosa and theca cells, after ovulation. Studies have reported that progesterone is involved in the development and growth of uterine fibroids [1].

Uterine fibroids are the most frequently occurring hormone-dependent benign tumors in the smooth musculature of the uterus in females at sexual maturation [2]. GnRH agonists have been used as conventional therapy, although it has some side effects, such as hot flashes and bone loss because of the suppression of estrogen. Ulipristal acetate (UPA), a selective progesterone receptor modulator (SPRM), has been gaining attention for the treatment of uterine fibroids in recent years. SPRMs are known to mainly bind to the progesterone receptor (PR) and exert agonistic or antagonistic activity depending on the tissue.

They have been found to selectively inhibit the growth of uterine fibroid cells without affecting normal uterine smooth muscle cells [3]. Research has also shown that the molecular biological mechanism of SPRM in uterine fibroids has various effects, such as suppression of myoma cell proliferation, induction of apoptosis, suppression of angiogenesis, and reduction of cytosol [4, 5].

UPA effectively reduces the size of uterine fibroids, thereby maintaining the secretion of estrogen equivalent to the follicular phase, in symptomatic patients with heavy menstrual bleeding [6]. Therefore, it has a low frequency of side effects in these patients due to their low estrogen status. SPRMs have been observed to induce endometrial thickening, as detected by ultrasound, by maintaining endogenous estrogen secretion, a phenomenon referred to as a PRM-associated endometrial change (PAEC) [7]. PAECs are recognized as pathologically benign reversible changes [8]. Although theoretically they differ from endometrial hyperplasia and endometrial cancer, their physiological significance and mechanisms have not been fully elucidated.

The prevalence of endometrial cancer, a hormone-dependent malignant tumor similar to uterine fibroids, has been increasing worldwide [9]. Unopposed estrogens, which are relatively excessively secreted estrogens uninfluenced by progesterone antagonism, are involved in the development of endometrial cancer, and endogenous and exogenous estrogen exposures are considered as risk factors for this. Progesterone is the key to hormone therapy for uterine fibroids in endometrial cancer. However, research into the application of UPA to treat endometrial cancer is scarce.

Moreover, many cancers have been reported to arise from inflammatory reactions induced by some proinflammatory chemokines [10]. In this study, the proinflammatory chemokines interleukin (IL)-6, IL-8, and oncostatin M (OSM) were focused. One study reported that 17β-estradiol promotes endometrial cancer proliferation and progression via activation of the IL-6 signaling pathway [11], and another revealed that OSM, a member of the IL-6 family, enhances endometrial cancer invasion and angiogenesis [12]. Research has established that OSM binding to the OSM receptor (OSMR) shows a wide variety of physiological actions via induction of the JAK (Janus kinase)/STAT (signal transducer and activator of transcription) and Ras/ERK (extracellular signal–regulated kinase) signaling pathways [13, 14]. These findings have generated interest in determining how UPA's maintenance of endogenous estrogen secretion influences inflammatory reactions in endometrial cancer. The aim of this research was to clarify the effects of UPA on endometrial cancer cells.

## 2 Materials and methods

### 2.1 Cell culture and chemical reagents

Ishikawa cells were cultured in Dulbecco's modified Eagle medium (DMEM; Gibco, Grand Island, NY, USA) containing 10% fetal bovine serum (Gibco), and maintained at 37 °C in 5% CO_2_. UPA in powder form was obtained from ASKA Pharmaceutical (Tokyo, Japan) and used as a chemical reagent. It was dissolved in 0.1% dimethyl sulfoxide, and an appropriate concentration thereof (0, 10, or 40 μM) was applied based on the previous reports [15, 16]. The estrogen receptor (ER) antagonist ICI 182,720 (hereinafter referred to as “ICI”) was also dissolved in 0.1% dimethyl sulfoxide, and its concentration (1 μM) was determined based on previous reports [17, 18]. DMEM/F-12, without phenol red (Thermo Fisher Scientific, Waltham, MA, USA) was used with the addition of ICI because phenol red has a weak estrogenic effect [19]. Mouse monoclonal antibody anti-β-actin (sc-47778), anti-p53 (sc-126), anti-Bax (sc-20067), and anti-Bcl-2 (sc-7382) were purchased from Santa Cruz Biotechnology (Dallas, TX, USA), whereas anti-cleaved PARP (cat. no. 5625) was obtained from Cell Signaling Technology (Danvers, MA, USA).

### 2.2 Cellular viability assay

Invasion and migration assays were performed as previously described [20, 21]. Briefly, Ishikawa cells at a density of 1 × 10^3^ cells/well treated with UPA (0, 10, or 40 μM) were seeded in 96-well microplate and incubated for 72 h. Cell viability assay was performed using Cell Counting Kit 8 (Dojindo, Tokyo, Japan) according to the instruction manual. All experiments consisted of triplicate wells for each sample and were repeated three times.

### 2.3 Colony formation assay

Colony formation assays were performed in accordance with the previous research [20]. Briefly, Ishikawa cells at a density of 1 × 10^3^ cells/well treated with UPA (0, 10, or 40 μM) were seeded in six-well plates, and then allowed to grow for 10 days. The number of colonies stained with 0.5% crystal violet (Sigma–Aldrich) was counted visually and calculated. All experiments, each consisting of triplicate wells for each sample, were repeated three times, and representative images were taken.

### 2.4 Invasion and migration assays

Invasion and migration assays were performed as previously described [21]. Briefly, we used Corning Matrigel Invasion Chamber 24-well plates and Corning BioCoat Cell Culture Inserts for 24-well plates (Corning, New York, NY, USA) following the instruction manuals. The invasion chamber, but not the control chamber, was rehydrated. Ishikawa cells (density, 0.25 × 10^3^) were then seeded with 500 μL of medium including serum on inserts and then mixed with medium with 40 μM UPA or without UPA including serum on wells. After 48 h of incubation, samples were stained with Diff-Quik solution. All experiments consisted of triplicate wells for each sample and were repeated three times.

### 2.5 Western blotting

The protein levels of Bax, cleaved PARP, Bcl-2, and p53 were detected by Western blotting based on previous reports [20, 21]. Ishikawa cells treated with UPA for 24h were used for measuring protein levels of Bax, Bcl-2, p53 and Ishikawa cells treated with UPA for 48h were used for measuring protein levels of cleaved PARP. The antibodies were diluted at the following concentrations: β-actin, 1:200; Bax, 1:200; cleaved PARP, 1:1000; Bcl-2, 1:200; p53, 1:500. All experiments consisted of triplicate wells for each sample and were repeated three times.

### 2.6 Caspase 3/7 activity detection

Caspase 3/7 activity was measured using the Caspase-Glo^Ⓡ^ 3/7 Assay System (Promega, Madison, WI, USA) according to the manufacturer's protocol. Ishikawa cells at a density of 3 × 10^3^ with UPA treatment (0, 10, or 40 μM) were seeded in 96-well microplates. Subsequently, 100 μL of Caspase-Glo^Ⓡ^ Reagent was added to each well and the well contents were agitated using a plate shaker at 400 rpm for 30 s. Plates were incubated at room temperature for 1 h, and the luminescence of each sample was then measured using a plate-reading luminometer (Wallac 1420 ARVO MX; Perkin Elmer, USA). All experiments consisted of triplicate wells for each sample and were repeated three times.

### 2.7 TUNEL assay

TUNEL assay was performed using the DeadEnd Fluorometric TUNEL System (Promega) according to the manufacturer's protocol. First, Ishikawa cells with serum-free DMEM were seeded on Lab-Tek Chamber Slides (Nunc, Rochester, NY, USA) and incubated for 24 h. The medium was replaced with fresh DMEM containing serum and different concentrations of UPA and then incubated for 72 h. The cells were fixed in 4% paraformaldehyde and then permeabilized in Triton X-100. DNase (cat. no. M6101; Promega) was added only for the positive control sample. Subsequently, the equilibration buffer was added and equilibrated in advance. After equilibration, green fluorescence nucleotides localized in apoptotic cells and rTdT enzyme were added and the chamber slides covered with a plastic coverslip were incubated for 1 h at 37 °C in the dark. The plastic coverslips were then removed, and the chamber slides were dipped in SSC twice. Next, ProLong Diamond Antifade Mountant with DAPI (Invitrogen, Waltham, MA, USA) was added to stain the nucleus of all cells incubated for 24 h at room temperature in the dark. The stained cells were visualized and measured using a confocal fluorescence microscope (FV10i; Olympus, Japan). All experiments consisted of triplicate wells for each sample and were repeated three times.

### 2.8 Cell cycle assay

Ishikawa cells at a density of 1 × 10^3^ cells/well were seeded and incubated with serum-free DMEM in six-well plates. After 12 h, the media were replaced with DMEM containing serum and an indicated concentration of UPA. The cells were further incubated for 72 h, stained with a BrdU Flow Kit (BD Biosciences), and then analyzed by flow cytometry. The experiments were repeated three times.

### 2.9 Real-time PCR

Total RNA was extracted using a High Pure RNA Isolation Kit (Roche, Basel, Switzerland) following the instruction manual. Quantitative real-time (qRT) PCR using a Power SYBER Green RNA-to-C_T_ 1-Step Kit (Thermo Fisher Scientific) and the 7500 Fast Real-Time PCR System (Thermo Fisher Scientific) were performed. The established primers of PR and its isoform PR-B were purchased from Invitrogen, whereas those of OSM, OSMR, IL-6, and IL-8 were obtained from BIORAD (Hercules, CA, USA). GAPDH was used as previously described [21]. The Ct values of PR, PR-B, OSM, and OSMR were calculated relative to GAPDH. All experiments consisted of triplicate wells for each sample and were repeated three times.

### 2.10 Statistical analysis

The graph bars are presented as the mean ± standard error of the mean (SEM). Statistical significance was determined using Student's t-test for paired comparison and one-way ANOVA with Bonferroni post hoc test for multiple comparisons using the GraphPad Prism 6 software (GraphPad, San Diego, CA) as appropriate. A p value less than 0.05 was considered statistically significant.

## 3 Results

### 3.1 UPA decreased cell number in a dose-dependent manner

**Figure 1:**
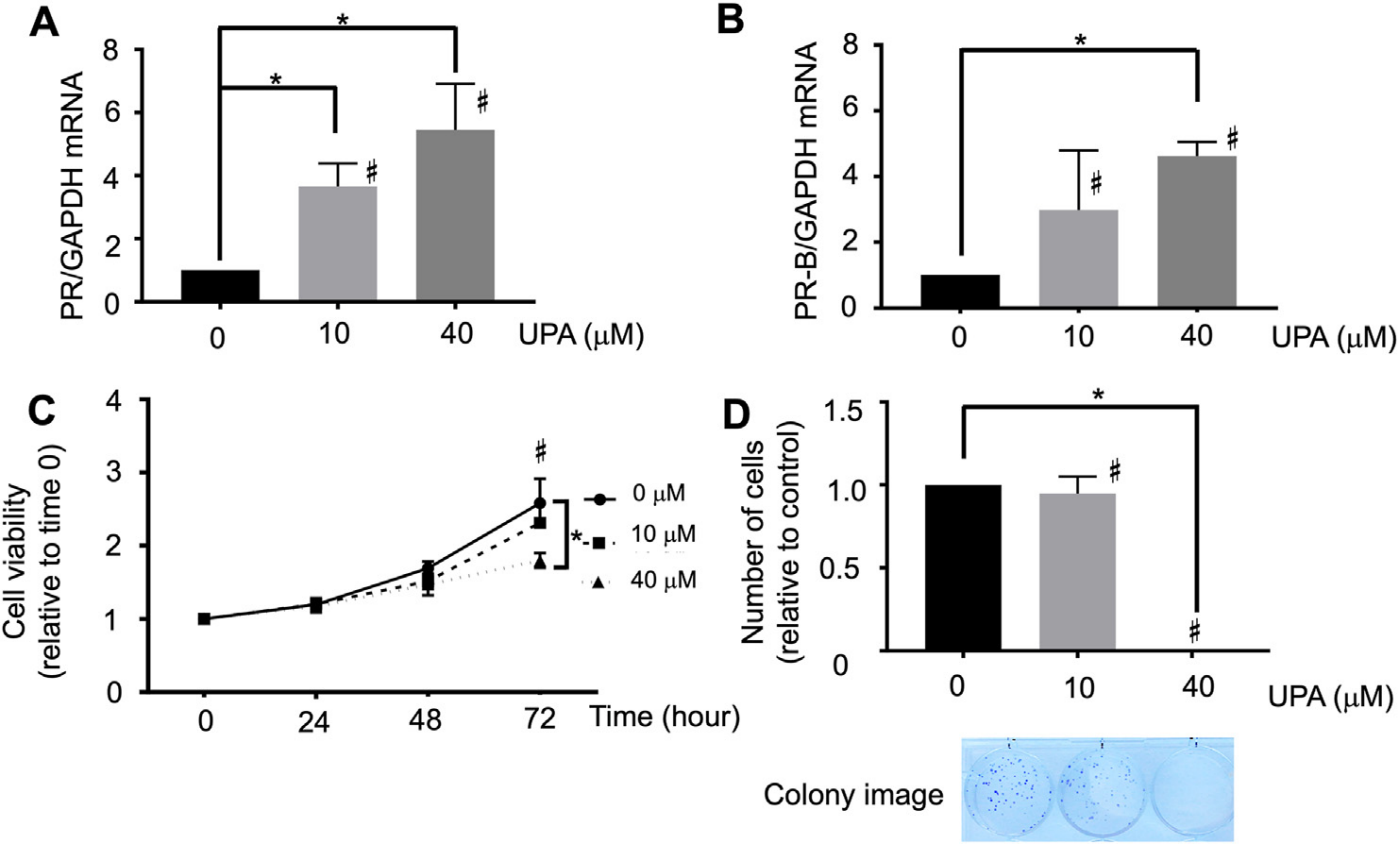
Expression of PR and PR-B messenger RNA levels and the effect of UPA on the growth of Ishikawa cells. (A, B) Ishikawa cells were treated with different concentrations of UPA and incubated for 72 h. The expression messenger RNA levels of PR and PR-B were determined using qRT-PCR and found to have increased in a dose-dependent manner. (C) UPA reduced Ishikawa cell proliferation in a dose-dependent manner. (D) Colony formation assay indicated that UPA reduced the number of colonies in a dose-dependent manner. A representative result of the colony formation assay is shown. All graph bars express ± SEM. ∗ denotes significantly different from control. (p < 0.05, ∗; one-way ANOVA with Bonferroni post hoc test).

Ishikawa cells were used because they recognize well-differentiated carcinoma cells and high expressions of PR according to a previous report [22]. qRT-PCR revealed that UPA upregulated the expression levels of PR and PR-B in a dose-dependent manner (Figure 1A, B). Moreover, cell viability assay demonstrated that UPA reduced cell viability (Figure 1C) and the number of colonies (Figure 1D) in a dose-dependent manner, particularly at the concentration of 40 μM.

### 3.2 UPA attenuated cell invasion and migration

**Figure 2:**
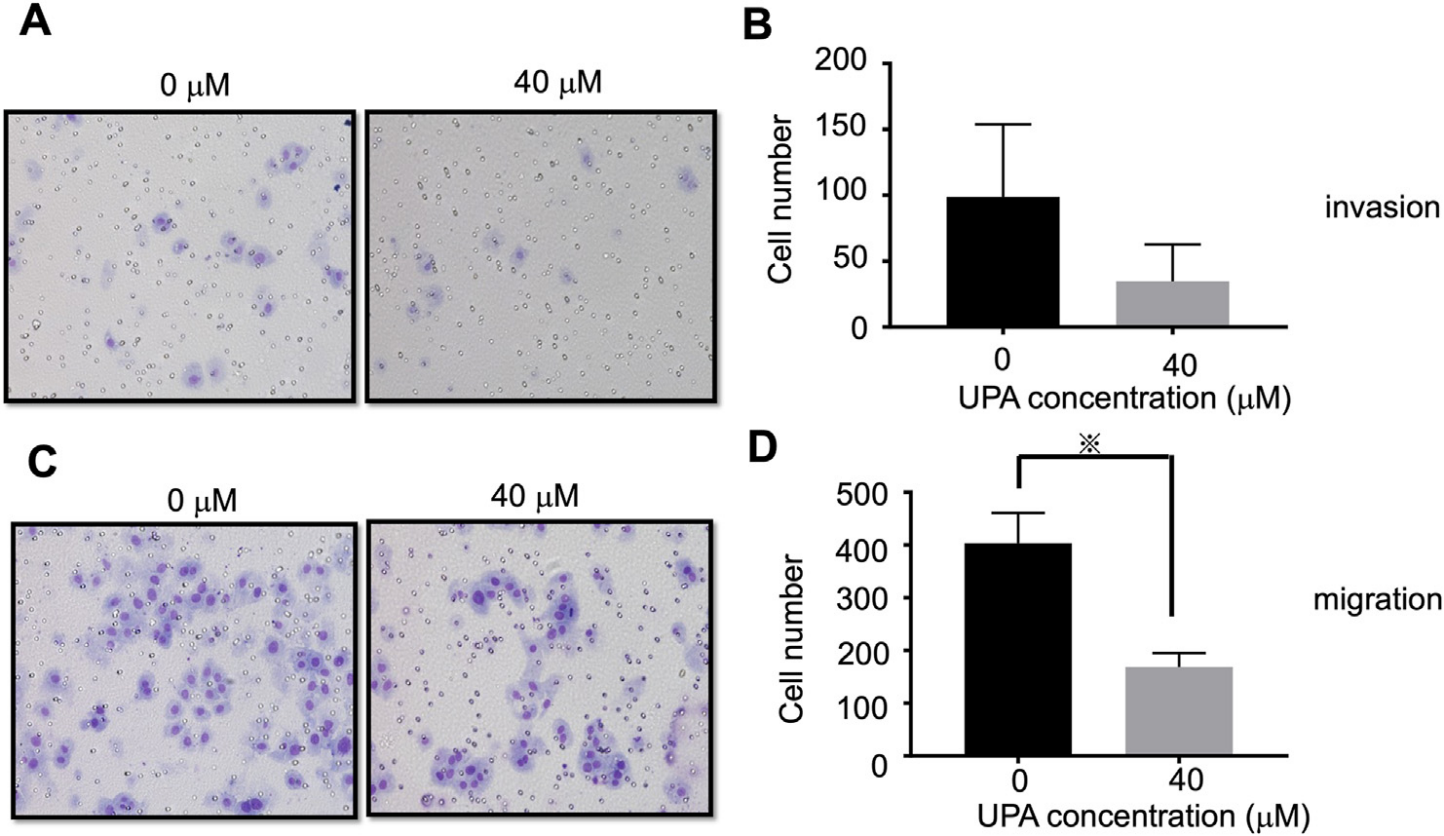
Invasion and migration assays. (A) Representative images of Ishikawa cells' transwell invasion after addition of 40 μM UPA for 48 h compared with negative control. (B) Representative graph of stained invasion cells visually counted indicating that UPA attenuated cell invasion ability. (C) Representative images of Ishikawa cells' transwell migration after addition of 40 μM UPA for 48 h compared with negative control. (D) Representative graph of stained migration cells visually counted indicating that UPA attenuated cell migration ability. All graph bars express ± SEM. ※ denotes significantly different from control. (p < 0.05, ※; Student's t-test).

Invasion and migration assays revealed that 40 μM UPA inhibited cell invasion and migration compared with samples without UPA, although a significant difference for invasion assay was not detected (Figure 2).

### 3.3 UPA induced apoptosis in a dose-dependent manner

**Figure 3:**
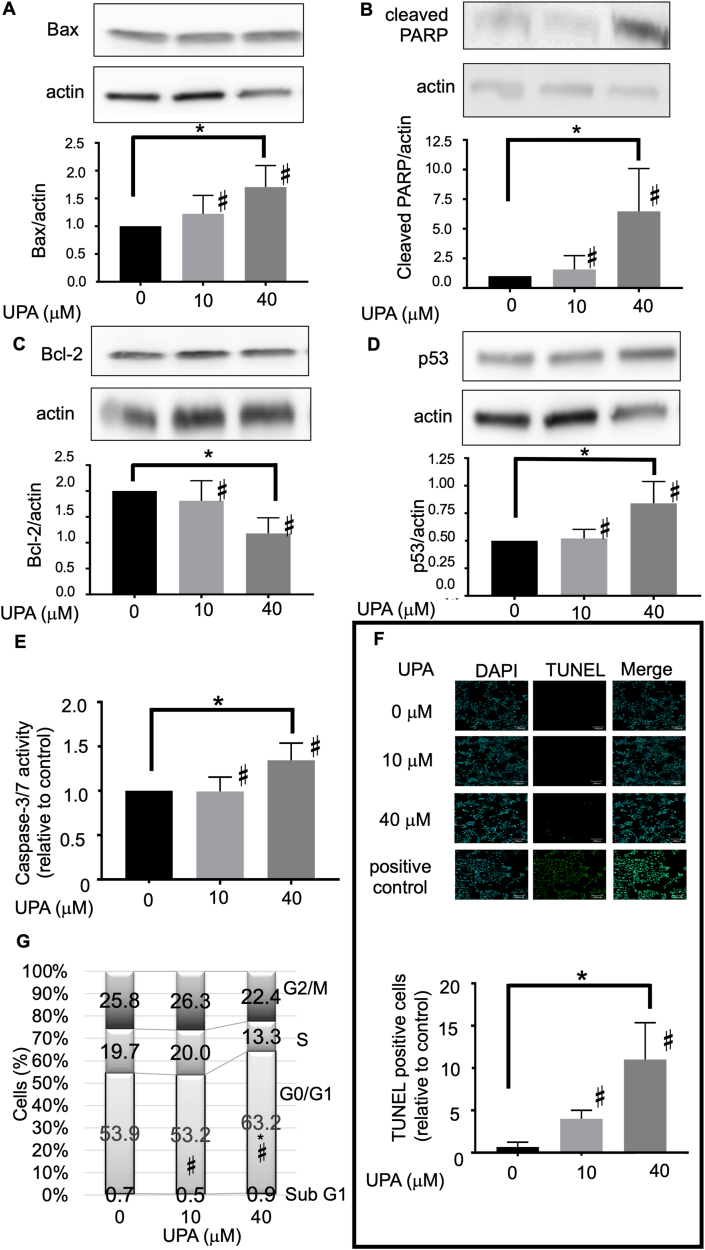
Various apoptosis and cell cycle assays. (A) UPA increased Bax expression in a dose-dependent manner. (B) UPA increased cleaved PARP expression in a dose-dependent manner. (C) UPA decreased Bcl-2 expression in a dose-dependent manner. (D) UPA increased p53 expression in a dose-dependent manner. (E) UPA treatment elevated caspase 3/7 activity in a dose-dependent manner. (F) TUNEL labeling and DAPI counterstaining in Ishikawa cells. UPA increased TUNEL-positive cells in a dose-dependent manner. (G) The proportion of cells arrested in the G_0_/G_1_ phase was significantly higher in cells treated with 40 μM UPA than in control cells. The results are expressed as the percentage of cells in each phase of the cell cycle (G_2_/M, S, G_0_/G_1_, Sub-G_1_). All graph bars express ± SEM. ∗ denotes significantly different from control. (p < 0.05, ∗; one-way ANOVA with Bonferroni post hoc test).

Western blotting using Ishikawa cells treated with UPA at 10 and 40 μM for 48 h and those treated without UPA showed that UPA dose-dependently increased the expression levels of Bax, cleaved PARP, and p53, a proapoptotic protein (Figure 3A, B, D and suppl Figure 1), but decreased the expression level of Bcl-2, an antiapoptotic protein (Figure 3C and suppl Figure 1). Subsequently, assay of caspase 3/7 activity as a functional endpoint of apoptosis cascade demonstrated that UPA dose-dependently upregulated it (Figure 3E). Furthermore, TUNEL assay, which was used to detect apoptotic cells undergoing fragmentation, confirmed that UPA treatment resulted in more TUNEL-positive cells compared with the control (Figure 3F). Finally, flow cytometry established that the proportion of cells arrested in the G_0_/G_1_ phase was significantly higher in cells treated with 40 μM UPA than in control cells (Figure 3G).

### 3.4 UPA alone induced the activation of OSM, OSMR, IL-6, and IL-8

**Figure 4:**
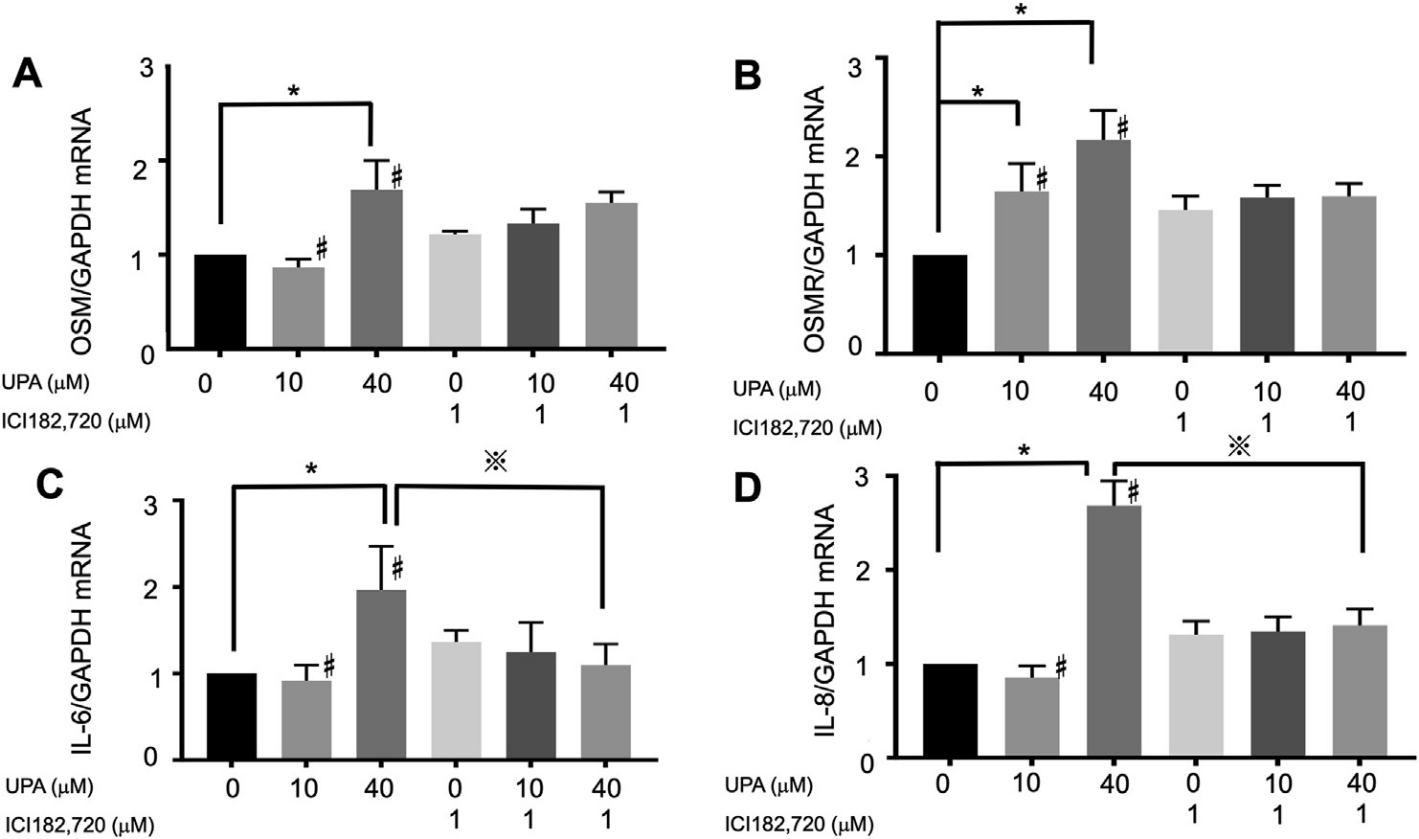
Expression of OSM, IL-6, and IL-8 in endometrial cancer cells by qRT-PCR using UPA and ICI. (A) UPA and ICI individually enhanced OSM expression, whereas UPA and ICI in combination tended to reduce it compared with stand-alone UPA at 40 μM. (B) UPA and ICI individually enhanced OSMR expression in a dose-dependent manner, whereas UPA and ICI in combination tended to reduce it compared with stand-alone UPA at 40 μM. (C) UPA and ICI individually enhanced IL-6 expression, whereas UPA and ICI in combination tended to reduce it compared with stand-alone UPA at 40 μM. (D) UPA and ICI individually enhanced IL-8 expression, whereas UPA and ICI in combination tended to reduce it compared with stand-alone UPA at 40 μM. All graph bars express ± SEM. ∗ denotes significantly different from control (p < 0.05, ∗; one-way ANOVA with Bonferroni post hoc test). ※ denotes significantly different from stand-alone UPA at 40 μM (p < 0.05, ※; Student's t-test).

qRT-PCR of Ishikawa cells treated with different concentrations of UPA for 72 h revealed that 40 μM UPA significantly enhanced the expression levels of OSM, OSMR, IL-6, and IL-8 compared with the negative control sample (Figure 4).

### 3.5 UPA and ICI in combination attenuated the proinflammatory cytokines

By itself, ICI enhanced the expression levels of OSM, OSMR, IL-6, and IL-8. However, the simultaneous use of UPA (40 μM) and ICI tended to downregulate the expression levels of these proinflammatory cytokines compared with stand-alone use of UPA (Figure 4).

## 4 Discussion

UPA binds to PR and provokes tissue-specific agonist, antagonist, or mixed agonist/antagonist activity depending on the tissue environment and ligand–receptor complex [23]. Several clinical studies have reported that the efficiency of UPA for the treatment of uterine fibroids is superior to that of leuprorelin acetate, a GnRH agonist [6]. However, criticism persists that UPA's maintenance of endogenous estrogen secretion could provoke PAECs. Although theoretically PAEC is different from endometrial hyperplasia and endometrial cancer, their physiological significance and the mechanism remains unclear. Thus, the physiological behavior of UPA in endometrial cancer cells were investigated in this study.

Herein PR and PR-B expression levels in Ishikawa cells were elevated by UPA in a dose-dependent manner. PR is a nuclear receptor that has two main isoforms, namely, PR-A (81 kDa) and PR-B (116 kDa), transcribed from different promoters of the same gene [24]. It has become apparent that UPA has an agonist effect in endometrial cancer based on the increased expression levels of PR and PR-B.

To investigate whether UPA inhibits endometrial cancer cell proliferation, cell viability and colony formation assays were performed and showed that UPA dose-dependently suppressed the growth of Ishikawa cells. Moreover, invasion and migration assays were performed to investigate the antitumorigenic properties of UPA and it was found that UPA dose-dependently decreased migration in Ishikawa cells. Furthermore, it has become apparent that UPA could induce apoptosis in a dose-dependent manner, as demonstrated by Western blotting, caspase 3/7 assay, flow cytometry analysis, and TUNEL assay. The results confirmed that UPA provoked apoptosis in not only uterine fibroids but also endometrial cancer cells. These findings indicate that UPA inhibits cell growth, migration, invasion, and activation of apoptosis in endometrial cancer cells. Medroxyprogesterone acetate (MPA), a progestin drug, was previously used in a patient with stage IA endometrial cancer and found to be efficient [25, 26], although thrombosis, a severe adverse event of MPA, poses a problem regarding its use. Hence, UPA might be a viable alternative treatment to MPA for patients with endometrial cancer who hope to preserve fecundity.

Changes in proinflammatory cytokines in Ishikawa cells treated with UPA were investigated and it was demonstrated that 40 μM UPA significantly upregulated the expression levels of OSM, OSMR, IL-6, and IL-8 compared with the negative control sample. OSM, a member of the IL-6 family of cytokines, binds to OSMR and then can either promote or inhibit the growth of various cancer cells [27, 28]. These results suggest that OSM performs different biological behaviors depending on the tumor cell type. In addition, Zhu et al. [12] reported that high OSM expression is positively correlated with tumor stage, histological grade, myometrial invasion, advanced stage, and lymph node metastasis in endometrial cancer tissues based on immunohistochemical analysis.

Estrogen produced by androgen through the action of aromatase also modulates the inflammatory response to develop cancer in postmenopausal women [29]. Additionally, the effect of UPA and ICI as an ER antagonist with suppressed estrogen secretion based on the above report were investigated. The present study demonstrated that only ICI challenged the proinflammatory cytokines in Ishikawa cells compared with untreated controls. Several studies have reported that ICI inhibits endometrial cancer cell growth [30, 31, 32], and inflammatory cells are recognized to promote carcinogenesis [10]. Nevertheless, data on the association between ICI and inflammatory cells are not available in the literature.

Next, the combination of 40 μM UPA and ICI tended to downregulate the expression of the proinflammatory cytokines compared with stand-alone UPA treatment at 40 μM. UPA is known to maintain endogenous estrogen secretion, and this hormonal dynamic is merited under the treatment of uterine fibroids because UPA could reduce the volume of uterine fibroid without climacteric symptoms. However, current results clearly indicated that UPA might cause inflammation in endometrial cancer cells. It can be speculated that the essential physiological properties of PAEC is partially attributed to this UPA function, which possibly open a new insight that the maintenance of estrogen secretion, which is a characteristic of UPA, might enhance the inflammatory effect and might induce carcinogenic function in endometrial cancer. Hence, simultaneous use of UPA and an ER antagonist for the treatment of endometrial cancer might be more desirable. On the other hand, UPA at the lower concentration of 10 μM did not remarkably induce inflammation compared with 40 μM UPA. It can be speculated that a relative estrogen excess due to a progesterone agonist at certain levels would trigger an inflammatory reaction.

This study has several limitations. First, the pathophysiology of PAECs has not been fully elucidated. PAECs are characterized by three pathological features: cystically dilated glands, nondeciduate compact stroma, and stroma with areas of prominent vascularity [7]. In line, UPA affects not only the endometrial epithelium but also the endometrial stroma. However, the behavior of UPA only in epithelial cells (Ishikawa cells) were investigated. Second, the combination of UPA and ICI did not suppress inflammation in a dose-dependent manner compared with UPA alone. Lastly, the present study uses only cancerous single cells as Ishikawa cells and not conduct co-culturing with non-cancerous cell lines, therefore, not compared to non-cancerous cell lines established therapeutic effects on Ishikawa cells.

These limitations highlight the need for further research into the molecular biological behavior of UPA under co-culture of epithelial and stroma cells and that on other cell lines.

In summary, the effects of UPA in endometrial cancer cells became evident: (1) UPA inhibits cancer cell growth via activation of apoptosis and (2) the combination of UPA and ICI attenuates inflammatory activity, but UPA alone provokes it. These findings indicate that UPA is a promising adjuvant therapy in endometrial cancer, particularly for patients who are considering fertility preservation.

## Declarations

### Author contribution statement

Ranka Kanda: Conceived and designed the experiments; Performed the experiments; Analyzed and interpreted the data; Wrote the paper.

Yuko Miyagawa: Performed the experiments; Analyzed and interpreted the data.

Osamu Wada-Hiraike: Conceived and designed the experiments; Analyzed and interpreted the data; Contributed reagents, materials, analysis tools or data; Wrote the paper.

Haruko Hiraike: Conceived and designed the experiments; Analyzed and interpreted the data.

Kazunori Nagasaka: Conceived and designed the experiments; Analyzed and interpreted the data; Contributed reagents, materials, analysis tools or data.

Eiji Ryo; Tomoyuki Fujii; Yutaka Osuga; Takuya Ayabe: Contributed reagents, materials, analysis tools or data.

### Funding statement

This work was supported by Grant-in-Aid for Scientific Research from the Japanese Ministry of Education, Science, and Culture (18K09248, 21K09489), 10.13039/100009619Japan Agency for Medical Research and Development (20gk0210018h0003) and 10.13039/501100003478Ministry of Health, Labour and Welfare (19FB1001).

### Data availability statement

Data will be made available on request.

### Declaration of interests statement

The authors declare no conflict of interest.

### Additional information

No additional information is available for this paper.

## Appendix A Supplementary data

Supplementary Figure revise Expression status of apoptosis-related genes. Various apoptosis-related factors were analyzed by Western blot, and these data show full blots. These blots demonstrate (A) Bax, (B) cleaved PARP, (C) Bcl-2, (D) p53 expressions. As described in materials and methods, the data obtained 24 h after UPPA exposure were used. Etoposide (Eto) served as a positive control for the induction of apoptosis. Supplementary Figure revise
