# Supplementary material for: Ulipristal acetate simultaneously provokes antiproliferative and proinflammatory responses in endometrial cancer cells
Source: Heliyon. 2021 Dec 29;8(1):e08696. doi: 10.1016/j.heliyon.2021.e08696 (PMC8749191; doi:10.1016/j.heliyon.2021.e08696)
Supplement: Supplementary Figure revise — Expression status of apoptosis-related genes. Various apoptosis-related factors were analyzed by Western blot, and these data show full blots. These blots demonstrate (A) Bax, (B) cleaved PARP, (C) Bcl-2, (D) p53 expressions. As described in materials and methods, the data obtained 24 h after UPPA exposure were used. Etoposide (Eto) served as a positive control for the induction of apoptosis. [file mmc1.pptx]

## Slide 1
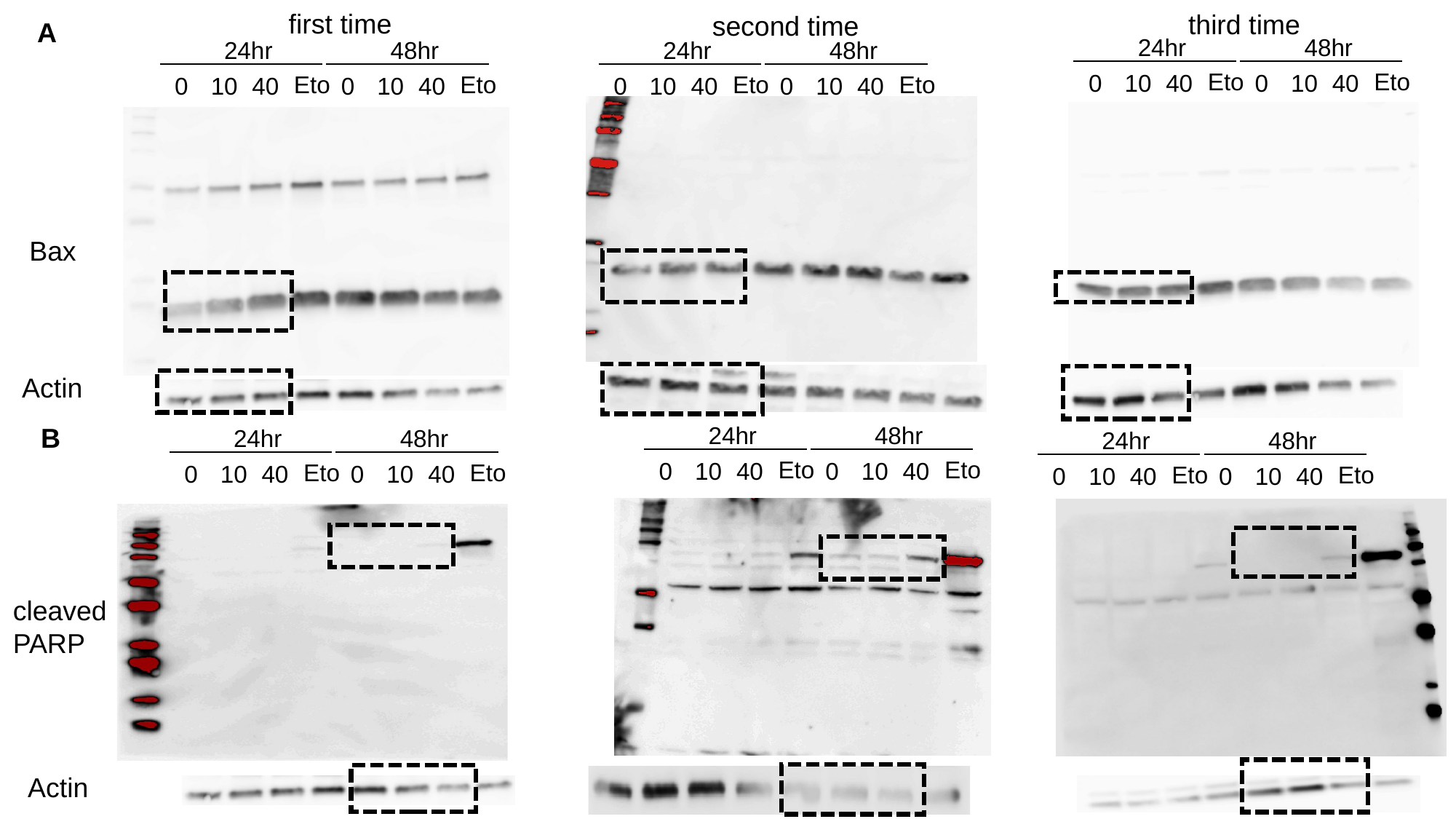

first time
third time
second time
A
24hr
48hr
Eto
Eto
0
10
40
0
10
40
24hr
48hr
Eto
Eto
0
10
40
0
10
40
24hr
48hr
Eto
Eto
0
10
40
0
10
40
Bax
Actin
24hr
48hr
Eto
Eto
0
10
40
0
10
40
B
24hr
48hr
Eto
Eto
0
10
40
0
10
40
24hr
48hr
Eto
Eto
0
10
40
0
10
40
cleaved PARP
Actin

## Slide 2
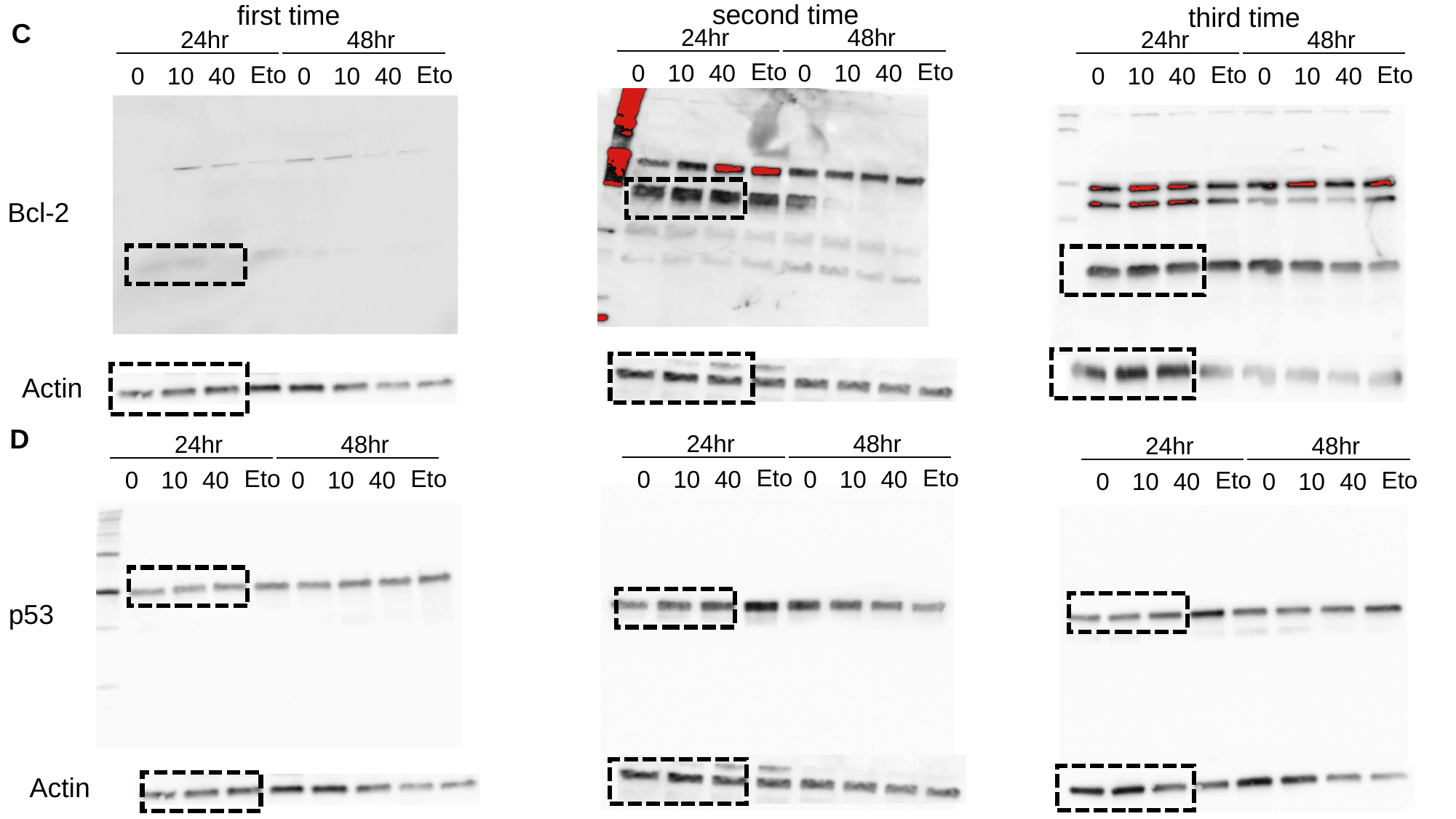

second time
first time
third time
C
24hr
48hr
Eto
Eto
0
10
40
0
10
40
24hr
48hr
Eto
Eto
0
10
40
0
10
40
24hr
48hr
Eto
Eto
0
10
40
0
10
40
Bcl-2
Actin
D
24hr
48hr
Eto
Eto
0
10
40
0
10
40
24hr
48hr
Eto
Eto
0
10
40
0
10
40
24hr
48hr
Eto
Eto
0
10
40
0
10
40
p53
Actin
20.2.5
